# Supplementary material for: Multi-contrast anatomical subcortical structures parcellation
Source: eLife. 2020 Dec 16;9:e59430. doi: 10.7554/eLife.59430 (PMC7771958; doi:10.7554/eLife.59430)
Supplement: Transparent reporting form [file elife-59430-transrepform.docx]

***eLife’s* transparent reporting form**

We encourage authors to provide detailed information *within their submission* to facilitate the interpretation and replication of experiments. Authors can upload supporting documentation to indicate the use of appropriate reporting guidelines for health-related research (see [EQUATOR Network](http://www.equator-network.org/ \t _blank)), life science research (see the [BioSharing Information Resource](https://biosharing.org/)), or the [ARRIVE guidelines](http://www.plosbiology.org/article/info:doi/10.1371/journal.pbio.1000412) for reporting work involving animal research. Where applicable, authors should refer to any relevant reporting standards documents in this form.

If you have any questions, please consult our Journal Policies and/or contact us: [editorial@elifesciences.org](mailto:editorial@elifesciences.org).

**Sample-size estimation**

- You should state whether an appropriate sample size was computed when the study was being designed
- You should state the statistical method of sample size computation and any required assumptions
- If no explicit power analysis was used, you should describe how you decided what sample (replicate) size (number) to use

Please outline where this information can be found within the submission (e.g., sections or figure legends), or explain why this information doesn’t apply to your submission:

This submission presents a tool for automated labeling of brain regions in MRI, thus the sample size (number of subjects to be labeled) is irrelevant.

**Replicates**

- You should report how often each experiment was performed
- You should include a definition of biological versus technical replication
- The data obtained should be provided and sufficient information should be provided to indicate the number of independent biological and/or technical replicates
- If you encountered any outliers, you should describe how these were handled
- Criteria for exclusion/inclusion of data should be clearly stated
- High-throughput sequence data should be uploaded before submission, with a private link for reviewers provided (these are available from both GEO and ArrayExpress)

Please outline where this information can be found within the submission (e.g., sections or figure legends), or explain why this information doesn’t apply to your submission:

The leave-one-out validation experiment was performed as a benchmark during the development of the tool, thus representing our best achievable performance. Note however that the developed method requires no parameter tuning, and that no statistical tests were performed for this experiment. The lifespan validation experiments were performed with the final developed tool. Both experiments used the same input data (technical replicates), and all measurements were included in the statistical analysis. An additional experiment used a secondary open access data set, including all measurements.

**Statistical reporting**

- Statistical analysis methods should be described and justified
- Raw data should be presented in figures whenever informative to do so (typically when N per group is less than 10)
- For each experiment, you should identify the statistical tests used, exact values of N, definitions of center, methods of multiple test correction, and dispersion and precision measures (e.g., mean, median, SD, SEM, confidence intervals; and, for the major substantive results, a measure of effect size (e.g., Pearson's r, Cohen's d)
- Report exact p-values wherever possible alongside the summary statistics and 95% confidence intervals. These should be reported for all key questions and not only when the p-value is less than 0.05.

Please outline where this information can be found within the submission (e.g., sections or figure legends), or explain why this information doesn’t apply to your submission:

Statistical analysis was only used to assess potential biases over the atlas size and lifespan in Figures 6 and 8. For Figure 6, the statistics compared measures obtained with atlases with Welch's t-test, using N=106 data points. For Figure 8, the statistical tool used was linear regression performed with ordinary least squares (OLS), with N=210 data points, and slope indicated when significantly different from 0 (as assessed by a F-test).

(For large datasets, or papers with a very large number of statistical tests, you may upload a single table file with tests, Ns, etc., with reference to sections in the manuscript.)

**Group allocation**

- Indicate how samples were allocated into experimental groups (in the case of clinical studies, please specify allocation to treatment method); if randomization was used, please also state if restricted randomization was applied
- Indicate if masking was used during group allocation, data collection and/or data analysis

Please outline where this information can be found within the submission (e.g., sections or figure legends), or explain why this information doesn’t apply to your submission:

Subjects were grouped into six age groups (18-30, 31-40, 41-50, 51-60, 61-70, 71-80) for the experiments presented in Figures 6 and 7, and three age groups (18-40, 41-60, 61-80) for Table 4. No other grouping was performed.

**Additional data files (“source data”)**

- We encourage you to upload relevant additional data files, such as numerical data that are represented as a graph in a figure, or as a summary table
- Where provided, these should be in the most useful format, and they can be uploaded as “Source data” files linked to a main figure or table
- Include model definition files including the full list of parameters used
- Include code used for data analysis (e.g., R, MatLab)
- Avoid stating that data files are “available upon request”

Please indicate the figures or tables for which source data files have been provided:

All numbers used to generate Figures 3, 4, 6, 7 and 8 are publicly available on the University of Amsterdam's FigShare: https://doi.org/10.21942/uva.12452444
